# Spectral phase measurement of a Fano resonance using tunable attosecond pulses

**DOI:** 10.1038/ncomms10566

**Published:** 2016-02-18

**Authors:** M. Kotur, D. Guénot, Á Jiménez-Galán, D. Kroon, E. W. Larsen, M. Louisy, S. Bengtsson, M. Miranda, J. Mauritsson, C. L. Arnold, S. E. Canton, M. Gisselbrecht, T. Carette, J. M. Dahlström, E. Lindroth, A. Maquet, L. Argenti, F. Martín, A. L'Huillier

**Affiliations:** 1 Department of Physics, Lund University, PO Box 118, SE-22100 Lund, Sweden; 2 Departamento de Química, Módulo 13, Universidad Autónoma de Madrid, 28049 Madrid, Spain; 3 Department of Synchrotron Radiation Instrumentation, Max IV Laboratory, Lund University, PO Box 118, SE-221 00 Lund, Sweden; 4 Department of Physics, AlbaNova University Center, Stockholm University, SE-106 91 Stockholm, Sweden; 5 Laboratoire de Chimie Physique-Matière et Rayonnement, Université Pierre et Marie Curie, 11, Rue Pierre et Marie Curie, 75231 Paris Cedex 05, France; 6 Instituto Madrileño de Estudios Avanzados en Nanociencia (IMDEA-Nanociencia), Cantoblanco, 28049 Madrid, Spain; 7 Condensed Matter Physics Center (IFIMAC), Universidad Autónoma de Madrid, 28049 Madrid, Spain

## Abstract

Electron dynamics induced by resonant absorption of light is of fundamental importance in nature and has been the subject of countless studies in many scientific areas. Above the ionization threshold of atomic or molecular systems, the presence of discrete states leads to autoionization, which is an interference between two quantum paths: direct ionization and excitation of the discrete state coupled to the continuum. Traditionally studied with synchrotron radiation, the probability for autoionization exhibits a universal Fano intensity profile as a function of excitation energy. However, without additional phase information, the full temporal dynamics cannot be recovered. Here we use tunable attosecond pulses combined with weak infrared radiation in an interferometric setup to measure not only the intensity but also the phase variation of the photoionization amplitude across an autoionization resonance in argon. The phase variation can be used as a fingerprint of the interactions between the discrete state and the ionization continua, indicating a new route towards monitoring electron correlations in time.

## Abstract (web summary)

Resonant absorption of light in atoms can lead to autoionization, whose probability exhibits a Fano intensity profile. Here, the authors use attosecond pulses and weak infrared radiation to study the phase variation of the photoionization amplitude across an autoionization resonance in argon.

## Graphical abstract

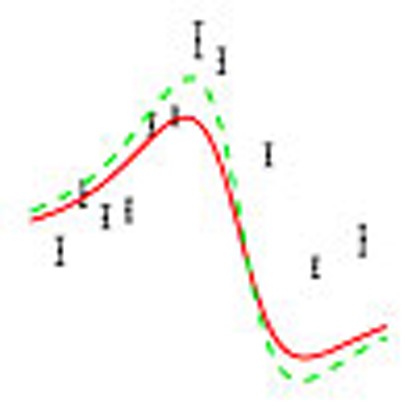

The development of table-top attosecond-duration sources in the extreme ultraviolet (XUV) spectral range has opened up possibilities of accessing the electron dynamics by pump–probe experiments. In recent experiments, photoionization delays have been measured using attosecond pulses combined with an infrared probe field in a variety of systems, from gas phase to solid-state samples^1^,^2^,^3^,^4^,^5^,^6^,^7^.

Photoionization dynamics is strongly affected by the presence of resonances. When a highly excited bound state is coupled to an open ionization channel, autoionization may occur via electron correlation. Interference between the direct and the autoionizing pathways leads to the characteristic asymmetric Fano profiles in the photoionization cross-section^8^,^9^, which have been extensively measured using synchrotron radiation^10^,^11^.

Studying and even controlling this interaction has been a major goal of attosecond science since the early days and several methods have been developed to this end^12^. Attosecond streaking was used for the first time-resolved measurement of Auger decay in Kr^13^, as well as for investigating autoionizing states in He^14^, while the more recent transient absorption technique was applied to studies of autoionization in Ar^15^ and He^16^,^17^. In the so-called reconstruction of attosecond beating by interference of two-photon transitions technique, trains of attosecond pulses combined with infrared probing allow for phase measurements, for example, in the vicinity of bound^18^ and autoionizing states^19^. Autoionizing resonances have also been studied using a single harmonic and a delayed infrared probe^20^, as well as by XUV pump/XUV probe spectroscopy^21^. This topic has stimulated vigorous theoretical activity^22^,^23^,^24^,^25^,^26^,^27^,^28^,^29^.

In this study, we present an interferometric study of photoionization of argon in the proximity of the 3*s*^2^3*p*^6^→3*s*^1^3*p*^6^4*p* autoionizing resonance (Fig. 1a) using a coherent XUV comb of odd-order harmonics of a tunable fundamental field. A synchronized, weak infrared probe field stimulates two-photon ionization, where, in addition to absorption of an XUV photon, an infrared photon is either absorbed or emitted, giving rise to sidebands in the photoelectron spectrum at energies corresponding to the absorption of an even number of infrared photons. Quantum interference between the pathways involving two neighbouring harmonics leads to oscillation of the sideband signals as a function of pump/probe delay^30^. The phases of the oscillations for sidebands 16 and 18 strongly depend on the detuning of harmonic 17 from the resonance. Our experimental measurements and theoretical calculations show that the spectral phase of an ionizing wavepacket is strongly distorted by the presence of a quasi-bound state and exhibits a non-trivial (different from *π*) variation. We give an interpretation for the phase variation, which reflects the interaction between the continuum 3*p*^−1^*ɛs*,*d* and the quasi-bound 3*s*^−1^4*p* states.

## Results

### Experimental results

Photoelectron spectra in the kinetic energy range between 4 and 20 eV, corresponding to photoionization with harmonics 13–23, were recorded as a function of the delay between the XUV and the infrared pulses (see Methods for details). When the laser wavelength is such that the 17th harmonic is detuned to be far off the resonance, the delays corresponding to the sideband maxima depend linearly on the electron energy. This dependence arises mainly from the intrinsic chirp of the attosecond pulses^31^. It was estimated for each excitation wavelength by linearly interpolating the maxima of sidebands 14, 20 and 22, and found to be on average equal to 0.16 rad (30 as) eV^−1^. The data presented in the following are corrected for this effect. The intensity of the probe beam was kept well below 10^12^ W cm^−2^, to suppress processes involving absorption or emission of more than one infrared photon, thus allowing us to neglect the influence of the probe field on the resonance.

In Fig. 2a, we extract the sidebands of the experimental photoelectron spectrum, using an energy of harmonic 17 close to the resonance. Clearly, the maxima of sidebands 16 and 18 are shifted in opposite directions. The photoelectron peaks are broadened due to the XUV and infrared field bandwidths, the spectrometer resolution and the spin–orbit splitting (0.17 eV), which is not resolved in the experiment. Figure 2b shows theoretical results obtained by using the method outlined below. To extract the phase of the oscillation, the sideband signal was fitted to an interference equation 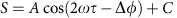 where 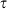 is the time delay between the XUV and the infrared pulses, *ω* the infrared frequency, Δ*φ* is the phase of the oscillation, *A* its amplitude and *C* a constant offset. A Fourier analysis of the oscillation was also performed, to verify that no component with a frequency higher than 2*ω*, due to higher-order processes, was present. No sideband signal was observed beyond XUV/infrared temporal overlap, thus indicating that the quasibound state is not or weakly populated in this interaction^27^.

Figure 3 presents the key results of this work, with the ionization signal (Fig. 3a) and the phase variations of sidebands 16 (Fig. 3b) and 18 (Fig. 3c) as a function of the photon energy of harmonic 17. The black symbols are the experimental results, while the red solid and green dashed lines show theoretical results obtained by using a recently developed two-photon resonant model^27^. The photoionization signal due to harmonic 17 shows the characteristic behaviour of a window resonance. The phase variation across the resonance, which is almost 0.6 rad, is asymmetric, with a bias towards positive values for sideband 16 and negative values for sideband 18.

### Theoretical calculations

**Figure 1:**
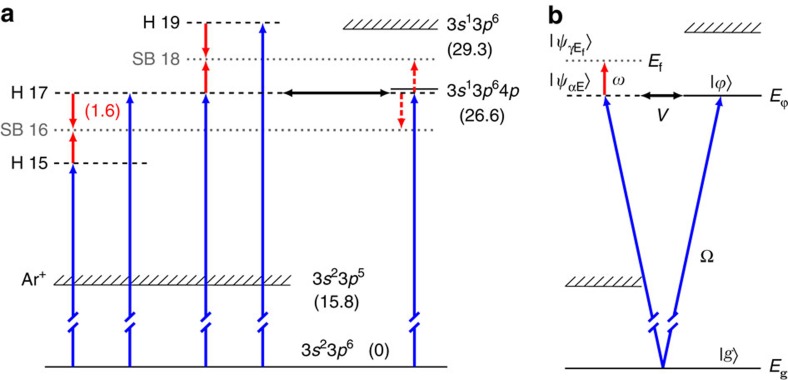
Energy diagrams. (**a**) Ar energy diagram showing the states, channels and processes involved in the present work. The blue arrows represent ionization at different harmonic frequencies. The red arrows denote absorption or stimulated emission of infrared photons. Energies (in eV) are indicated in parentheses. The energy of harmonic 17 can be tuned across the 3*s*^−1^4*p* resonance, which decays by autoionization (black arrow). The processes indicated by the red dashed arrows are found to be weak, as discussed below. (**b**) Energy diagram presenting some of the theoretical notations used in this work. 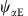 denote continuum wavefunctions with energy *E* and angular momentum *α*, 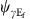 are final continuum wavefunctions with angular momentum *γ* and energy *E*_f_. The excited bound state is described by the wavefunction 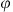 and the energy 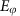. *g* and *E*_g_ are the ground-state wavefunction and energy. Ω and *ω* are the XUV and infrared photon energies. *V* represents the configuration (Coulomb) interaction.

**Figure 2:**
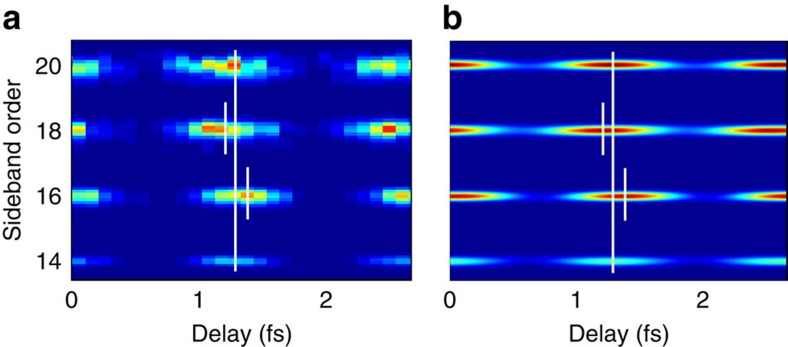
Photoelectron spectra as a function of delay. (**a**) Photoelectron signal for sidebands 14, 16, 18 and 20 as a function of delay between the XUV radiation and the infrared field. The laser wavelength is chosen such that the central energy of harmonic 17 is 26.63 eV, in close resonance with the 3*s*^−1^4*p* state. The photoelectron signal at the harmonic frequencies has been removed for clarity and the results have been corrected for the chirp of the attosecond pulses. The short white lines indicate the position of sidebands 16 and 18, while the long lines join the maxima of sidebands 14 and 20. The position of the maxima of sidebands 16 and 18 is strongly affected by the presence of the resonance, towards positive delays for sideband 16 and in the opposite way for sideband 18. (**b**) Theoretical calculations using the approach presented in the main text agree well with the experimental results.

Our theoretical derivation follows the well-known formalism developed by Fano^9^, to account for the interaction between the continuum channels and the quasi-bound state, and generalizes it to include the influence of a weak infrared field, in the perturbative limit^27^. Here we briefly describe the essence of this model. The notations are indicated in Fig. 1b. The transition matrix element for two-photon ionization involving the absorption of a harmonic photon Ω and the absorption/emission of an infrared photon from the ground state *g* to a final continuum state 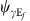, labelled by its energy *E*_f_ and angular channel *γ*, can be written as


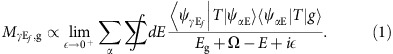


An integral sum is performed over all non-resonant (field-free) intermediate states 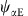 in the discrete or continuum spectrum (energy *E*) for each of the allowed angular momentum channels *α*; 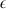 is an infinitely small positive quantity and *T* is the dipole transition operator. The multi-channel character of the transition is essential here, as for argon the ^1^P^o^ autoionizing states that converge to the 3*s*^−1^ threshold decay through two independent channels: 3*p*^−1^*ɛs* and 3*p*^−1^*ɛd*. Finally, *γ* indicates any of the three possible final states with either *S* or *D* symmetry, [3*p*^−1^*ɛp*]_*S*_, [3*p*^−1^*ɛp*]_*D*_ and [3*p*^−1^*ɛf*]_*D*_, whose contributions must be summed incoherently to obtain the overall sideband intensity^5^,^32^. Equation (1) uses intermediate states 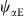, which are eigenfunctions of an unperturbed Hamiltonian *H*_0_.

To include the Coulomb interaction (*V*) between the continuum states and the bound state 3*s*^1^3*p*^6^4*p* described by a wavefunction 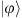 and with an energy 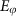, we first transform 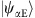, 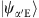 with *α* and *α*′ referring to the *s* and *d* continua, respectively, into interacting 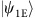 and non-interacting 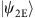 according to:


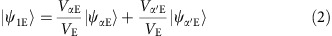



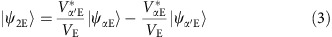


with |*V*_E_|^2^=|*V*_αE_|^2^+|*V*_α′E_|^2^, 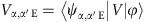. Obviously, 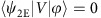, while 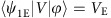. This transformation allows us to simplify the problem to that of a bound state interacting with a single non-resonant continuum channel. We now diagonalize the full Hamiltonian (*H*=*H*_0_+*V*) in the 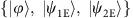 basis, which leads to 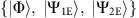 expressed as


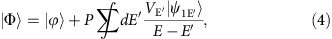



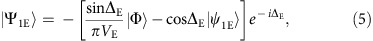



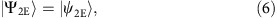


with *P* denoting the Cauchy principal value. The quantity Δ_E_ is the phase shift of 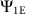 with respect to 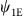 and *E*_Φ_ is the energy of the resonance:


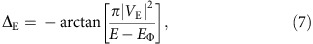



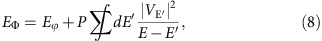


Introducing the parameter *q* and the reduced energy 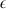



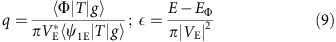


the one-photon transition matrix elements become


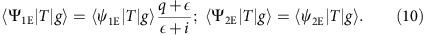


The one-photon ionization cross-section is the sum of the absolute squares of these matrix elements, the non-interacting channel contributing only by a smooth background to the Fano profile. If one neglects radiative transitions from the resonance to the continuum (dashed arrows in Fig. 1a), it can be shown, after some manipulations, that the two-photon transition matrix element (equation (1)) that includes now the effect of the resonance can be written as


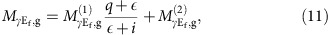


where 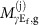 (*j*=1, 2) has a similar expression as equation (1) with the intermediate wavefunctions now equal to 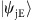 and where *q*, 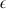 are calculated at the energy *E*_g_+Ω. All of these quantities smoothly vary with energy and the resonant effects are contained in the ratio 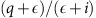.

**Figure 3:**
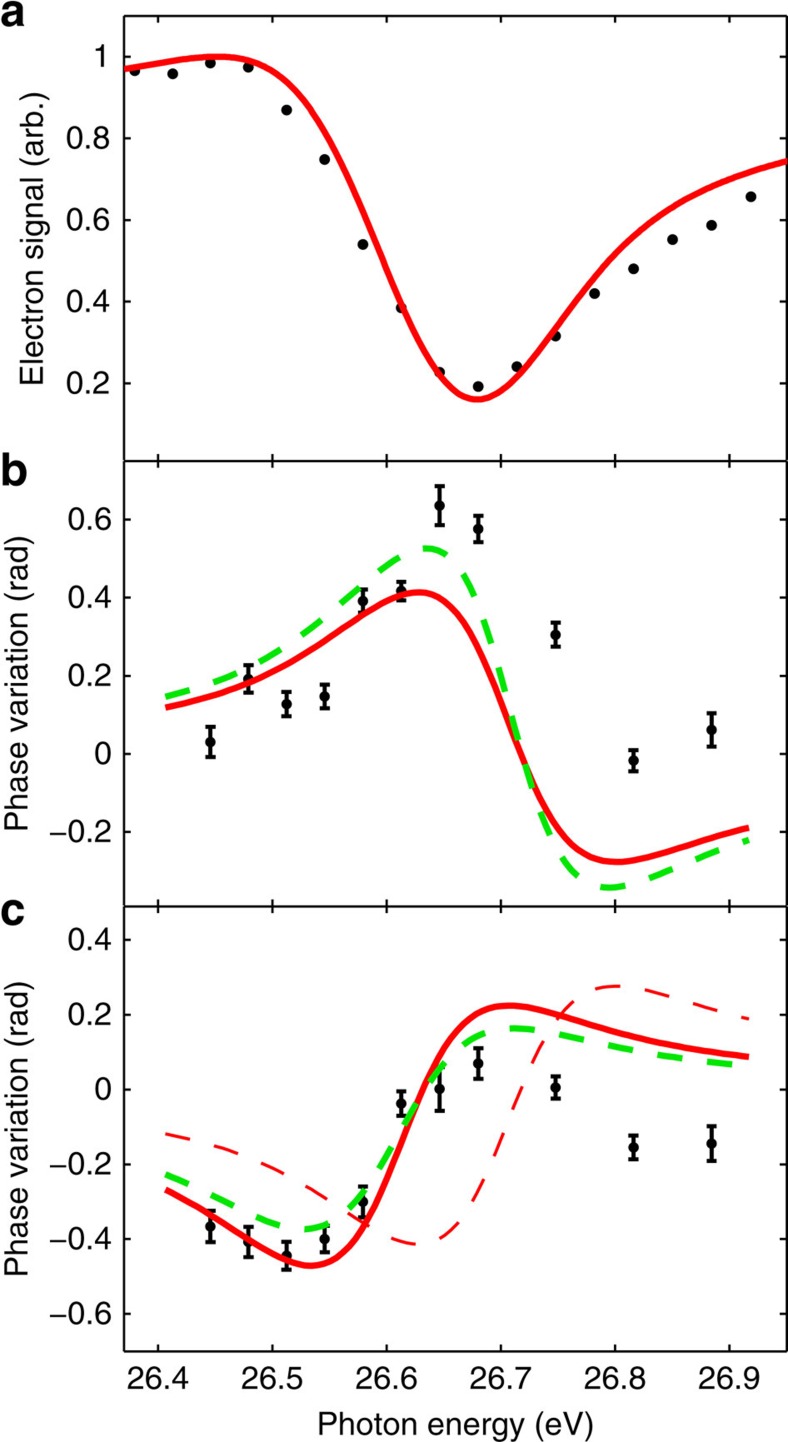
Intensity and phase variation of the photoionization amplitude. (**a**) Photoionization signal as a function of harmonic 17 photon energy. The black symbols denote experimental results and the red curve calculations. Phase variation of sideband 16 (**b**) and sideband 18 (**c**) as a function of the energy of harmonic 17. The theoretical results are indicated by the red solid line, whereas the experimental results are shown by the black symbols. The error bars represent the statistical uncertainty (1 s.d.) of the phase determined by fitting sideband oscillations. The thin dashed red line in **c** is the opposite of the red line for sideband 16, which is close to the corresponding results for sideband 18, apart from an energy shift. This energy shift can be attributed to the influence of the partly ionized medium on the probe laser wavelength. The green dashed lines correspond to calculations including the processes indicated with dashed arrows in Fig. 1a. The agreement between theory and experiment is not significantly improved by taking these processes into account.

Details of our calculations, which also include finite pulse effects^27^, are presented in the Methods section. Figure 3 shows our results as a solid red line. The agreement with the experimental data, both for the ionization signal due to harmonic 17 and for the phase variation of sidebands 16 and 18, is convincing, especially on the low-energy side of the resonance. In principle, the phase variation of sidebands 16 and 18 should be of opposite sign, as the resonance affects the path corresponding to absorption of an infrared photon for sideband 16, whereas it affects the path where an infrared photon is emitted for sideband 18. The red dashed line in Fig. 3c is the opposite of the variation of sideband 16. It is quite close to the red line representing the variation of sideband 18, apart from an energy shift. This shift comes from the fact that the harmonic energies are slightly higher than a multiple of the probe photon energy, owing to the ionization-induced blue shift of the fundamental field in the generation cell and is accurately reproduced by our model when realistic pulse parameters are used (see Methods). The comparison between theoretical results for infinite-duration pulses and the present experimental data allows us to estimate the influence of the XUV spectral width on the resonance features: The spectral width of the resonance is broadened by a factor 2 compared with monochromatic radiation, while the total phase variation is reduced by a factor of ∼3 and the resonance profile remains similar.

We also examined the influence of other possible processes induced by the interaction of the resonant state with the infrared field, as represented by the dashed arrows in Fig. 1a. These processes can be included by changing *q* into a complex parameter 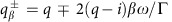, with the + sign for sideband 18 and the − sign for sideband 16 (ref. 27). 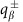 approaches *q* when the couplings between the intermediate, quasi-bound part of the (radial) wavefunction and the final continua are weak. The best agreement between the predictions of this extended model and the experimental measurements is found when *β* is very close to zero (*β*=0.005) as indicated by the green dashed curves in Fig. 3.

## Discussion

**Figure 4:**
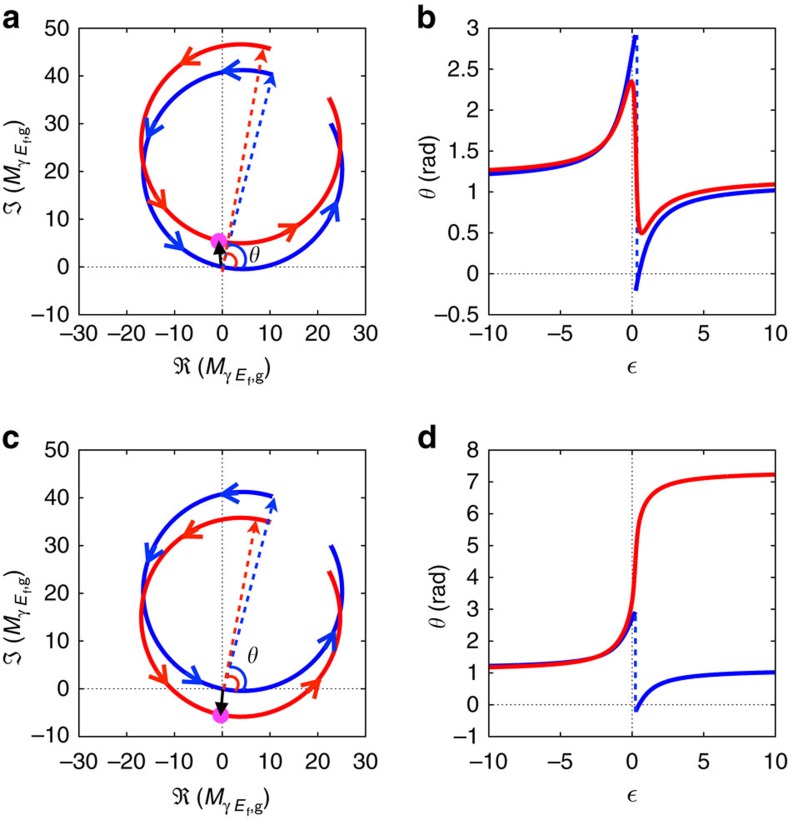
Influence of multiple channels. (**a**) Complex plane representation of 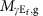 (red circle), its resonant 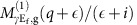 (blue circle) and non-resonant 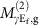 (black arrow and magenta dot) components (see equation (11)); *θ* represents here the initial phase at the reduced energy 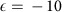. The final state has angular momentum *L*=0, 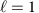 and no finite pulse effects are included. (**b**) Phase variation of 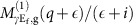 (blue line) and 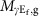 (red line) across the resonance. The resonant contribution exhibits a *π* jump across the resonance, while the phase variation of the total amplitude is less than *π*. (**c**,**d**) Similar representations for the opposite phase of the non-resonant contribution; 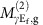 (black arrow and magenta dot) is replaced by its complex conjugate. The red circle (**c**) now crosses the horizontal axis and the phase variation (**d**) is 2*π*.

The measured phase profile across the autoionizing resonance can be interpreted by considering the multichannel nature of the problem, explicit in the matrix element 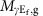 (equation (11)). Figure 4a shows the trajectory of 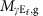 (angular momentum *L*=0, 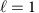) in the complex plane, as well as its resonant and non-resonant contributions, as the reduced energy varies from −10 to 10. The resonant contribution describes counterclockwise a circle that passes through the origin for 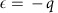. In the absence of a contribution from the non-resonant channel, the phase of 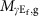 follows the phase of the Fano profile, 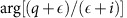. The phase increases first steadily with energy, then drops discontinuously by *π* when 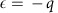, to increase again thereafter, until it attains its original value (blue line in Fig. 4b). For small values of *q* as is the case in the present work (*q*=−0.25 (refs 10, 11)), the *π* phase jump occurs close to 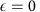 and the phase variation is almost symmetric. In the presence of a non-interacting channel, the phase variation across the resonance will in general differ from *π*. In the present case, the non-resonant complex amplitude moves the circular trajectory away from the origin, as indicated by the phase vectors (phasors) in Fig. 4a. As a result, the phase of the total amplitude varies smoothly across the resonance (red line in Fig. 4b) and by a total amount less than *π*. In other cases, the non-resonant amplitude could shift the circle towards the origin instead of away from it (see example in Fig. 4c), in such a way that the trajectory of the total amplitude encircles the origin. In that case, the total phase variation is close to 2*π* (Fig. 4d). The dynamics of the photoelectron wavepacket created by absorption of XUV radiation with energy close to the resonance is strongly affected by it and quite different in the two cases.

Our method complements attosecond streaking^13^,^14^ and attosecond transient absorption spectroscopy^15^,^16^,^17^ in several ways. Both methods use single attosecond pulses and a relatively intense infrared field, either overlapping or delayed relative to the XUV pulse. Attosecond streaking measurements determine directly in the temporal domain autoionization lifetimes, while attosecond transient absorption spectroscopy allows for measurement and control of the resonance lineshape. In refs 16, 33, a correspondence is made between the *q*-parameter and the phase shift of the temporal dipole response of the excited system, which can be controlled by a delayed few-cycle laser pulse and which differs from the spectral phase variation determined in the present work. Here we use the spectral resolution of the high-order harmonic coherent frequency comb combined with the possibility to tune the laser wavelength, as well as a weak infrared dressing field, to determine the spectral phase of the continuum wavepacket close to a Fano resonance. The deviation from a *π*-jump measured experimentally combined with our theoretical analysis indicate that the spectral phase brings new information about the electron–electron interaction when the quasi-bound state is coupled to more than one continuum channel.

In summary, we have measured the distortion of the phase of the continuum induced by the coupling with an autoionizing state, using an interferometric method based on two-photon ionization (see also a different, but related method in ref. 34). The experimental results are well reproduced by a calculation based on *ab-initio* parameters for the configuration interaction. Our tunability range covers from about half the spectrum at 15 eV to all of it from 50 up to 100 eV, using harmonics generated in Ne to get higher photon energy. This range could be extended both at low and high energy by using an (tunable) optical parametric amplifier and/or by mixing radiation at other frequencies. Our method allows us to study a number of resonances in rare gases including He and Ne, as well as to probe processes involving excitation of shallow core and inner valence shells in many alkalis, metals and metalloids. It thus provides a useful tool for characterization (both in phase and amplitude) and ultimately control of electron interactions.

## Methods

### Experimental method

We used an amplified titanium sapphire laser system, which delivers 5 mJ, 20 fs pulses, centred around 800 nm, at a repetition rate of 1 kHz. The laser system includes two acousto-optical programmable dispersive filters, capable of shaping the phase and amplitude of the pulses. A dazzler is placed in the stretched oscillator beam. It can be used to compensate the nonlinear phase accumulated by the pulse in the amplifier system, but also to restrict the bandwidth of the seed pulse. A mazzler is placed inside the cavity of a regenerative amplifier, where it is used to counter the gain narrowing by diffracting the spectral components that experience a high gain out of the cavity. In this way, we were able to achieve a bandwidth of up to 100 nm at the output of the laser chain and a tunability range of up to 50 nm for longer pulses with a reduced bandwidth of ∼50 nm. The pulses were directed into an actively stabilized Mach–Zehnder interferometer similar to that described in ref. 35. In one of the arms, high-order harmonics were generated in a pulsed gas cell, followed by a 200-nm-thick aluminum foil, which removed the leftover fundamental field. The harmonic and the infrared pulses were collinearly recombined and focused into a diffusive gas target in the interaction region of a magnetic bottle electron spectrometer.

### Calculations

To determine the unitary transformation defined in equations (2) and (3), we used *ab-initio* complex partial transition amplitudes^36^. The strength of the transition amplitudes between the non-resonant components of the intermediate and final continuum channels were estimated in the plane-wave approximation^37^. To compare with the experimental results, it is necessary to account for the finite duration of the attosecond pulse train, estimated to be ∼12 fs and of the fundamental infrared field (25 fs)^27^,^38^. In addition, we included a small blue shift of the generating fundamental field relative to the probe field. This effect arises from ionization-induced dispersion effects in the generating medium.

## Authors contribution

M.K., D.G. and D.K. have carried out the experiments and performed the analysis of the data. A.J.-G. has carried out the calculations. All these four authors have equally contributed to the article. E.W.L., S.B. and J.M. have provided the main experimental setup. M.L., M.M. and C.A. have provided an advanced tunable laser system. D.G., M.G., S.E.C., T.C., J.M.D., E.L., A.M., L.A., F.M. and A.L. have contributed to the theoretical ideas, formalism and calculations. M.G., L.A. and F.M. have contributed to the writing of the manuscript. M.K. and A.L. have written the main part of the manuscript.

## Additional information

**How to cite this article:** Kotur, M. *et al.* Spectral phase measurement of a Fano resonance using tunable attosecond pulses. *Nat. Commun.* 7:10566 doi: 10.1038/ncomms10566 (2016).
